# On Milk

**Published:** 1842-04

**Authors:** 


*On Milk.*—At the meeting of the *Acad. of Sciences*, on the 6th June, M. Donne communicated some observations on milk. Before the researches of the author, it was generally believed that milk is acid on being drawn from the breast; but M. Donne proved that the fresh milk of the human female, and of the female ass, is alkaline; as for the milk of the cow some doubts still remained, because it is almost neutral, and has the curious property both of turning blue test-paper red, and of restoring the blue color of paper that has been reddened by an acid. Sometimes, indeed, cow's milk is acid; we then discover by the microscope, that the milk-globules, instead of floating separately in the fluid, (as is the case in healthy milks) are collected together in little masses, and it seems probable that this change depends on the commencing coagulation of the caseum. In a recent case where it was a matter of the utmost importance to obtain perfectly pure milk, the author found this alteration in the milk of a cow which had been selected for her healthy appearance. The milk was acid, and the globules agglomerated.

At the previous meeting M. Donne communicated the results of some experiments with milk, which he had performed on various animals. Milk of different qualities was injected into the vessels and great cavities of dogs, horses, goats, frogs, &c. Milk of good quality had no bad effect when mixed with the blood; while, on the contrary, unwholesome milk gave rise to various accidents and sometimes occasioned death. *Am. Jour. Med. Science*, from *Prov. Med. Jour.*, June 19, 1841.

